# Co-Adaptation and the Emergence of Structure

**DOI:** 10.1371/journal.pone.0071828

**Published:** 2013-09-10

**Authors:** Robert Savit, Maria Riolo, Rick Riolo

**Affiliations:** 1 Department of Physics, University of Michigan, Ann Arbor, Michigan, United States of America; 2 Center for the Study of Complex Systems, University of Michigan, Ann Arbor, Michigan, United States of America; 3 Department of Mathematics, University of Michigan, Ann Arbor, Michigan, United States of America; University of Maribor, Slovenia

## Abstract

Co-adaptation (or co-evolution), the parallel feedback process by which agents continuously adapt to the changes induced by the adaptive actions of other agents, is a ubiquitous feature of complex adaptive systems, from eco-systems to economies. We wish to understand which general features of complex systems necessarily follow from the (meta)-dynamics of co-adaptation, and which features depend on the details of particular systems. To begin this project, we present a model of co-adaptation (“The Stigmergy Game”) which is designed to be as *a priori* featureless as possible, in order to help isolate and understand the naked consequences of co-adaptation. In the model, heterogeneous, co-adapting agents, observe, interact with and change the state of an environment. Agents do not, *ab initio*, directly interact with each other. Agents adapt by choosing among a set of random “strategies,” particular to each agent. Each strategy is a complete specification of an agent's actions and payoffs. *A priori*, all environmental states are equally likely and all strategies have payoffs that sum to zero, so without co-adaptation agents would on average have zero “wealth”. Nevertheless, the dynamics of co-adaptation generates a structured environment in which only a subset of environmental states appear with high probability (niches) and in which agents accrue positive wealth. Furthermore, although there are no direct agent-agent interactions, there are induced non-trivial inter-agent interactions mediated by the environment. As a function of the population size and the number of possible environmental states, the system can be in one of three dynamical regions. Implications for a basic understanding of complex adaptive systems are discussed.

## Introduction

Many of the most interesting biological and social systems fall into the category of complex adaptive systems (CAS), by which we mean systems of (generally) heterogeneous agents who co-adapt or co-evolve. The process of co-adaptation or co-evolution is one in which agents alter their strategies or natures (and consequently their actions) in response to the actions of other agents who are, in parallel, also altering their strategies and natures, and their subsequent actions. The environment (more generally, the context) experienced by an agent is, in part, endogenously produced by the actions of other agents, and it is that context which conditions the adaptations or the evolution of that agent. This strong population-wide endogenous feedback loop is one of the most basic underlying dynamics of CAS's. (To avoid awkward text, in the sequel, except where it may lead to confusion, we will often use the term “co-adaptation” to stand for “co-adaptation and/or co-evolution”. There will be times when we will want to distinguish between co-adaptation and co-evolution. Those will be clear in the text.)

Aside from co-adaptation, CAS's exhibit a number of other directly observable general features, albeit with variations. These include, for example, specific inter-agent interactions with various kinds of dynamics (e.g., mutualism, amensalism, etc.), as well as the existence (presumably emergence) of higher order structures or communities (eg. multi-celled organisms in biological systems or manufacturing firms with specialized workers in economic systems) which can themselves be thought of as agents at higher levels of organization. Other important common features have to do with the nature of the environment in which agents find themselves and their interactions with it, which therefore involve questions of niche formation and exploitation, habitat selection, etc. [1] [2] [3] [4].

Our notion is that the fundamental process of co-adaptation is of a different type from, and (in a logical sense) lies outside the universe of observed specific inter-agent dynamics, the interaction of agents with their environment, and the consequent emergence of higher order structures. We therefore consider co-adaptation (or co-evolution) as meta-dynamics. Because these meta-dynamics are so central to social or biological complex systems, it is important to ask what the observable consequences are of such meta-dynamics, absent any other specific assumptions about inter-agent dynamics. In asking this question we are well aware of the many confounding elements, contingencies and particular details of specific systems that may affect the observed inter-agent interactions and emergent structures. Nevertheless, it is important to understand what follows simply from co-adaptation, and what is dependent on other details of the system. That is, one of our overriding questions is the question of “universality” in co-adaptive systems.

In searching for universality in CAS's, it is not clear what quantities are candidates for universal behavior or, indeed, in what sense (e.g. quantitative or qualitative) one can expect to see universality. Nevertheless, the fact that some features do seem to be common across many different CAS's does suggest that the search for universality may bear fruit.

In this paper we report on the results of a study of a very simple model of co-adaptation which represents a first step in our project of understanding which features of CAS's flow from the meta-dynamics of co-adaptation, and which features are universal and in what sense. This model has been designed to be as simple and *a priori* as featureless as possible, the better to try to isolate and understand the naked consequences of co-adaptation. Furthermore, in this first version of the model, we restrict the agents' direct actions to actions on the environment. We shall see that identifiable agent-agent interactions are induced in this model, but they are all mediated by the environment. We will also see that an *a prior* random environment can become ordered as a result of the actions of the co-adapting population of agents. Because all interactions are mediated by the environment, we call this model the “Stigmergy Game”.

In the model, N adaptive agents interact with and modify the environment by inducing transitions among the E possible states in which the environment can find itself. When a given agent acts on the environment, he gains a payoff (positive, negative or neutral) and benefits (positive payoff) if, at the time he acts, the environment is in a certain subset of its E possible states. Different agents benefit from different environmental states. But the probability that the environment is in a particular state at a particular time depends on the cumulative actions of all agents. Thus there is a very strong feedback effect in that the wealth accrued by each agent depends on the way in which the environment is endogenously modified by the combined actions of all the agents.

The adaptive agents are heterogeneous. Each agent has available to it a set of strategies which are random in a sense that will be described below. The actions of an agent, as well as the rewards it accrues for its actions are dictated by one of the agent's (random) strategies. The adaptivity of the agent is embodied in the agent's ability to adopt different (random) strategies at different times, in reaction to the current structure of the environment which is the result of the cumulative action of all the agents.

As a function of N and E, we find three fairly distinct regions in systems with co-adaptive agents. In one region, the (initially disordered) environment takes on a strongly ordered form, with a subset of environmental states dominating. This region exhibits strong niche formation (i.e., the predominance of a subset of environmental states) and niche exploitation by agents (i.e., agents reap positive wealth from those predominating environmental states). In this region most agents do well at amassing wealth. In a second region, agents continue to amass positive wealth, but the environment has much less order (less than or equal to the environmental order produced by a set of non-adaptive agents). In the third region, agents amass only limited wealth, and the environment is also less ordered than in the non-adaptive case. This third region is distinguished from the other two by a kind of “thrashing” behavior in which agents continually change which strategies they use and are unable to sustain much long-lived coordination.

The rest of this paper is organized as follows. In the next section we will describe our computational model, and will also define a set of metrics that we will use to characterize the behavior of the model. In the results section we will present some results of numerical studies of the model. The paper ends with a discussion section which contains a discussion of the results, conclusions, and suggestions for future research.

## The Stigmergy Game

### A. Model Specification

The model studied in this paper consists of N co-adaptive agents. Agents do not interact directly with each other, but act on the environment, so that information about other agents is only transmitted stigmergically, that is, through the medium of the environment. The local environment can be in any one of E states. Agents act on the environment in random order. (We shall describe a version of this game in which agents act in a fixed order, elsewhere.) When an agent acts, he first observes the current state of the environment (say, e_1_) and then changes it to a new state, say, e_2_. (In this formulation of the models, the environment is passive. But in many cases, environments have their own non-agent (physical) dynamics and/or are composed of agents themselves and are therefore not passive. We will address this issue in future versions of our models.)

As a result of his action the agent receives a payoff which in this version of the game can be +1, −1 or 0. The agent's actions and consequent payoffs are dictated by a strategy, which is a look-up table of E rows and 3 columns, an example of which is shown in Table 1 below for E = 7. A strategy is a complete specification of actions and rewards for an agent given any circumstance he will encounter. The first column is a list of all E environmental states. When it is some agent's turn to act, he observes the current state of the environment and finds that state in the first column of his current strategy. The second column is a list of those states which result after the agent acts and the third column is a list of rewards for each possible action. The second and third columns are random (IID) lists, with the added constraint that the sum of the third column is zero, so that each strategy is reward neutral. Thus, if an agent using the strategy in Table 1 observed environmental state 4, he would turn that into state 7 and gain a payoff of +1 for that action. Each agent is endowed with S such randomly generated strategies (different sets of strategies for different agents). At a given point in the game, an agent uses its strategy which, had it played that strategy for all times in the past, would have resulted in the largest total payoff among all his strategies. If two or more strategies share the highest payoff, the agent chooses among them randomly. Thus, the agents are adaptive in that they can change their strategies at various points in the game in response to their own experience. (Here we discuss the simplest possible version of the game. We have also studied variations of this game including games in which strategy rankings are based on historically discounted accrued rewards. These variations do not materially alter our conclusions. They will be discussed in detail elsewhere.) It is important to stress that the agents' strategies in this game are random and reward neutral and so, *a priori*, any non-random structure or positive wealth generation in this game is likely to be attributable to the meta-dynamics of co-adaptation. (The effects of small sample random structure will be addressed below.)

**Table 1:** An example of a strategy table.

Current environmental state	New environmental state	Reward
1	5	+1
2	2	−1
3	4	0
4	7	+1
5	6	0
6	1	0
7	7	−1

### B. Metrics

There are many questions that can be asked of this model. A more extensive analysis of this model will be reported elsewhere, but here we concentrate on four metrics: 1. average rate of agent wealth accumulation, 2. standard deviation of wealth among agents in a game, 3. environmental order, and 4. a measure of how often agents switch their strategies. We will also briefly comment on induced inter-agent interactions in this model. These metrics will be sufficient to demonstrate the most striking aspects of the emergence of structure (including niche formation and exploitation) as a consequence of co-adaptation.

#### 1. Average rate of agent wealth accumulation

This is just the average over all agents of the net increase in an agent's wealth for each time the agent acts, averaged over some time window. This rate is between [1,−1] with a (non-adaptive) expectation value of zero. Specifically, the rate of wealth accumulation by an agent, i, during a time, T can be written
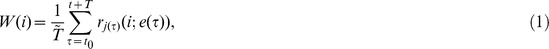
(1)Where the times during which agent i acts are denoted by τ and 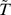 is the total number of times that agent i acts during the time interval [t_0_,t_0_+T]. We suppose that this i^th^ agent uses his j^th^ strategy at time τ, and r_j(τ)_(i;e(τ)) is the entry in the reward column of the j^th^ strategy associated with the (input) environmental state which the agent sees at time τ, e(τ). This can be rewritten as
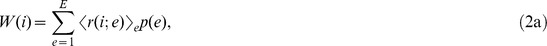
(2a)where p(e) is the probability for finding the environment in state e during the time T, and 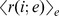 is the average value of the reward column of the strategies that agent i used over the time T, whenever that agent encountered environment e. If there are no correlations between the environment an agent sees and the strategy that the agent uses (which we expect to be the case for large T), then 2a becomes
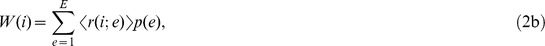
(2b)


In the special case that an agent uses only his j^th^ strategy over this time, 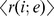 just becomes r_j_(i). Since 
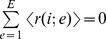
 (strategies are reward neutral), (2b) can also be written as
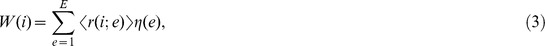
(3)where 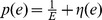. I.e., 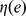 measures the deviation from a flat distribution of the probability of finding the environment in state e. Note from (3), that W(i) can be understood as a measure of an agent's ability to adapt and exploit niches. Contributions to W(i) are positive if an agent “likes” an environment, e, and if that environment occurs with probability greater than 1/E. Similarly, contributions to W(i) are also positive if an environmental state occurs with probability less than 1/E (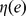
<0) and an agent “dislikes” that state(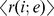
<0). W(i) can thus be thought of as an agent's success in the process of habitat selection, or niche exploitation. Note that since rewards and agent actions are embodied in the same strategies, the probability distributions p(e) are endogenously created by the agents themselves in acts of collective habitat construction. Thus, the extent to which the 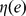 are non-zero can be thought of as a measure of the efficacy of the system at creating niches (niche formation) [3] [4]. Note also that if all 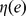 = 0, then all W(i) will be exactly zero (although the converse is not true). The wealth accumulation, averaged over agents is
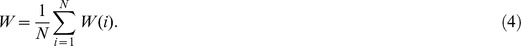
(4)


#### 2. Standard deviation of wealth accumulation

For a given run of the model, we compute the standard deviation of the rate at which wealth is accumulated by all agents over some time window. This is just
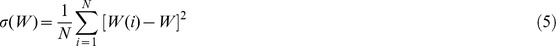
(5)


#### 3. Environmental order

As a measure of environmental order we use
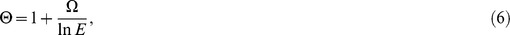
(6)where the environmental entropy is
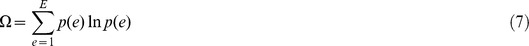
(7)and p(e) is the probability to find the environment in state e, averaged over some time window. If the environment is maximally disordered so that 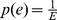 for all e, then 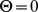. If the environment is maximally ordered so that 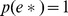 for some e* and 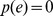 for all 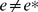, then 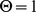.

The more the 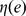 are non-zero the greater will be the environmental order. Thus, the environmental order can be thought of as a measure of the efficacy of the system at creating a subset of favored environmental states. We think of these environmental states as niches, since, typically, (as indicated in equation (3)) larger values of 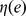 lead to greater agent wealth (although the relationship is not entirely monotonic, as we shall show below). The collective process of generating non-zero values of the 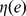 can be thought of as an abstract example of niche formation.

#### 4. Switching Rate

There are several possible metrics of switching rate which illuminate somewhat different features of the model. Here we take a very straight-forward one as a measure of switching rate, namely, the probability that an agent switches his strategy in a given time step. Since the agents act in random order, this probability represents a uniformly sampled average over all agents of the probability that an agent switches his strategy.

## Results

In the following figures we show results as a function of E and N for our four metrics, primarily (but not exclusively) for S = 64. These data represent averages over 32 runs (with different initial sets of random strategies) for each value of N, E and S. Each trial of the game was run for N×10^6^ time steps and the data for the last 10^4^ time steps was collected and analyzed. Note that in the S = 1 case each agent is endowed with only one (random) strategy and so there is no possibility of adaptation. This non-adaptive game is a base case for understanding the role of co-adaptation.

**Figure 1:**
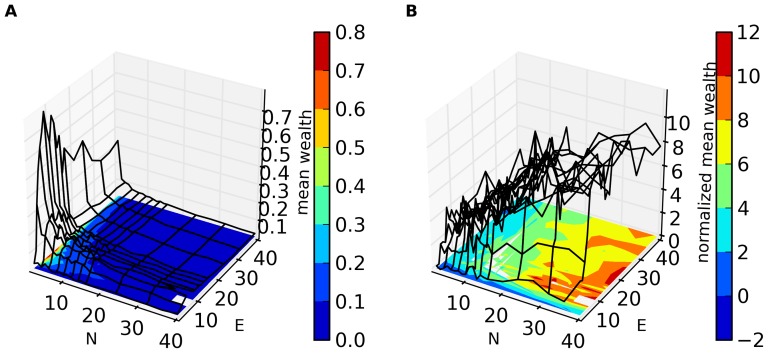
a. Mean wealth as a function of E and N for S = 64. Results shown here are averaged over 32 runs (with different initial sets of random strategies) for each value of N and E. Each trial of the game was run for N×10^6^ time steps and the data for the last N×10^4^ time steps was collected and analyzed. **1b. Q (** **equation 8** **) for mean wealth as a function of N and E.**

**Figure 2:**
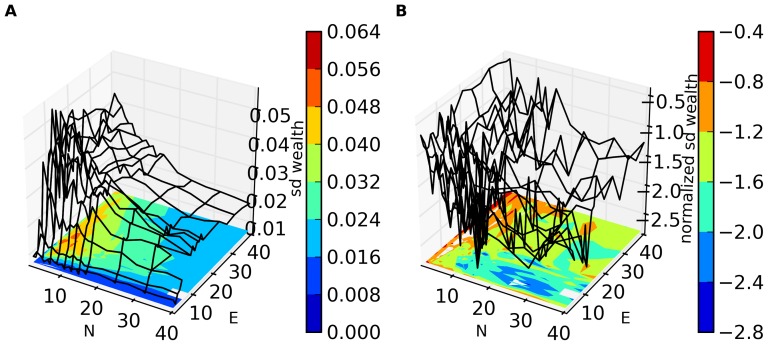
a. Standard deviation of the within-run agent wealth as a function of E and N for S = 64. Results shown are averaged over 32 runs (with different initial sets of random strategies) for each value of N and E. Each trial of the game was run for N×10^6^ time steps and the data for the last N×10^4^ time steps was collected and analyzed. **2b. Q (** **equation 8** **) for the standard deviation of within-run agent wealth as a function of N and E.**

**Figure 3:**
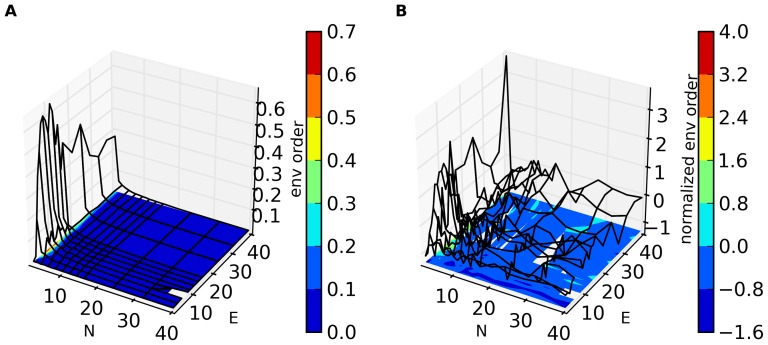
a. Environmental order (**equation 6**) as a function of E and N for S = 64. Results shown are averaged over 32 runs (with different initial sets of random strategies) for each value of N and E. Each trial of the game was run for N×10^6^ time steps and the data for the last N×10^4^ time steps was collected and analyzed. **3b. Q (** **equation 8** **) for environmental order as a function of N and E.**

**Figure 4:**
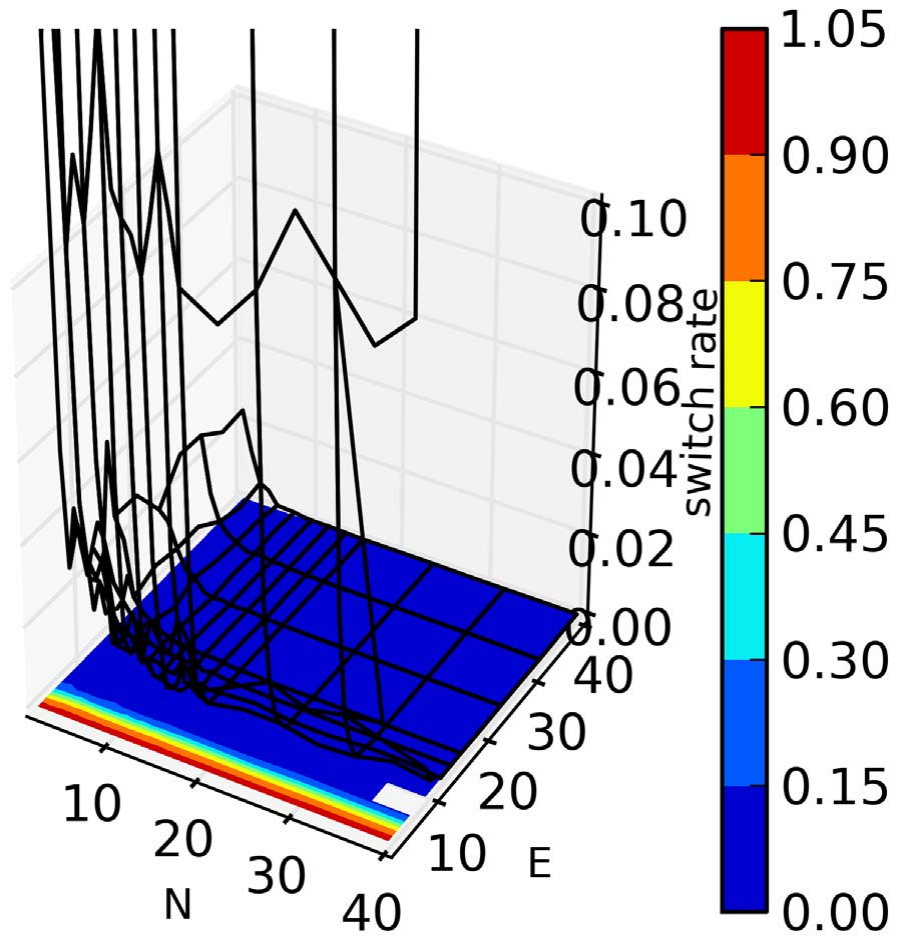
Switching probability as a function of E and N for S = 64. Results shown are averaged over 32 runs (with different initial sets of random strategies) for each value of N and E. Each trial of the game was run for N×10^6^ time steps and the data for the last N×10^4^ time steps was collected and analyzed.

In figures 1–4, we present an overview of the results for this system. In these figures, S = 64, and each of our four metrics is presented, as a function of E and N. In figures 1–3 the metrics are presented in two different formats. For each metric, Fig. a shows the value of the metric as a function of E and N, while Fig. b show that metric normalized with respect to the results found for the S = 1 case. Specifically, for a metric, M, we plot in Figs. b the following quantity:
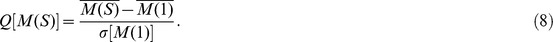
(8)Here M(S) is the mean value of the metric M in one run of the game played with S strategies, 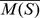, is the average of that quantity over several different runs of the game, and 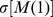 is the standard deviation of that metric over several different runs with S = 1. Q is therefore a measure of the degree to which the results in games with S>1 strategies (co-adaptive) differ from the results with S = 1 (no co-adaptation). The sign of Q is also important. The reason for considering this normalization is to more directly see the effects of co-adaptation (S>1). This is important because even with S = 1 there may be some small sample random effects, as we shall discuss in more detail below. Of course, there is no Fig. 4b since there is, by definition, no switching for S = 1.

Look first at Fig. 1, mean wealth. In Fig. 1a, we see that for all N and E, mean wealth is positive. Recall that each strategy is reward neutral so, absent the dynamics of co-adaptation, we expect mean wealth to be zero. (This is, in fact, the case for S = 1, when averaged over many runs.) For small N, mean wealth is high. There is also an interesting ridge around E = 6 which we shall briefly discuss in the discussion section, part D, below. In Fig. 1b we plot Q[mean wealth], and we see that relative to the S = 1 case, mean wealth tends to rise as N and E increase, although it is also high for small N. The systematic rise in Q with N is due to the fact that as N increases, deviations in small sample random effects become less pronounced so that the denominator in (8) becomes smaller. That is, it becomes increasingly difficult for non-adaptive agents to accumulate any positive wealth as the number of agents increases. For very small E, the numerator of (8) also decreases so in that case Q in Fig. 1b is small, but still positive.

In Fig. 2a, we plot the standard deviation of wealth among agents in a run. Here we see a relatively large standard deviation for small N (although in absolute terms rather small) falling as N increases. For low E the standard deviation of wealth is quite small. This is associated with the small value of mean wealth in this region (Fig. 1a): For small E, agents obtain only small positive wealth and there are no very wealthy agents. Interestingly, Fig. 2b shows that for all N and E, the standard deviation of wealth is smaller than in the non-adaptive case (S = 1). Q also tends to fall as E decreases and as N increases. Thus one persistent product of co-adaptation is that wealth tends to be more uniformly distributed among the agents: There are fewer (relatively) very poor or very wealthy agents.

Figs. 3 show environmental order. Recall that some degree of environmental order (some nonzero η(e)) is necessary for agents to obtain non-zero wealth. In Fig. 3a we see high order for small N, rapidly falling off as N increases. (It is worth remarking that in cases in which the environmental order is fairly large, this order develops over time. Typically, environmental order is close to zero near the beginning of a run, rising over time, but not necessarily monotonically, to a non-zero value. This will be discussed in detail elsewhere.) Fig. 3b is particularly interesting. Here we see that for larger E and N and for small E, the environment is typically *less* ordered than in the non-adaptive (S = 1) case. In Fig. 1b, on the other hand, we see that co-adaptivity results in larger average wealth gain than in the non-adaptive case in all regions, despite the fact that environmental order for large N, E and for small E is smaller than in the non-adaptive case. We shall discuss this further below.

An additional illuminating metric is the probability that an agent switches his strategy at some time step during which he acts. I.e., this is the probability that, at a given time step, an agent uses a strategy which is different than the one he last used. Fig. 4 shows this probability as a function of E and N, again for S = 64. Switching probability is very high for low E, independent of N and drops rapidly near E = 5. The switching rate is also relatively high for small N. We shall discuss these results below.

**Figure 5:**
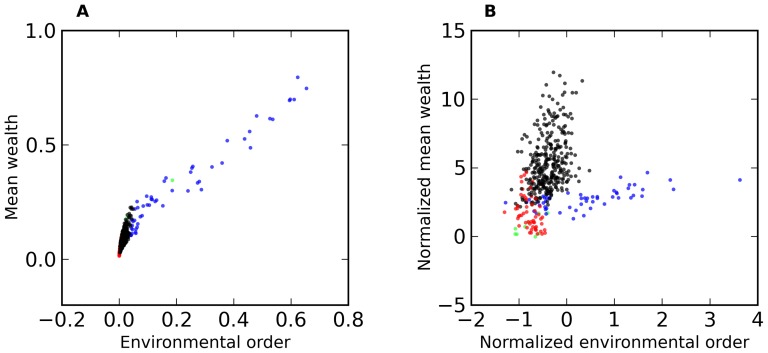
a. Mean wealth vs. environmental order, color coded according to E and N. Red is E≤5 and N>5, Blue is N≤5 and E>5, violet is N and E both ≤5, and black is N and E both >5. Data is the same as that used in Figs. 1–4. **5b. Q(mean wealth) vs. Q(environmental order).** Same coloring scheme as fig. 5a.

Figs. 1–4 suggest that, as a function of E and N, the system can be in one of three different regimes governed by different dynamics. To make this clearer, we present in Figs. 5 scatter plots of the results for different E and N (S = 64). Fig. 5a shows mean wealth vs. environmental order, and Fig. 5b shows Q(mean wealth) vs. Q(environmental order). The colors refer to the region of (E,N) space associated with that result. Blue is N≤5 (region A), black is N and E>5 (region B) and red is E≤5 (region C). (The few points that satisfy both N≤5 and E≤5 are colored violet.) (These region boundaries are only approximate, and in any case depend on S, as we shall discuss below.) In both these graphs the three regions are fairly distinct. Consistent with our understanding of the qualitative relationship between environmental order and mean wealth, Fig. 5a shows a positive slope in all three regions. However, the slope is markedly different for N≤5, than in the other two regions, as is the range of mean wealth and environmental order that the system achieves. In Fig. 5b, the three regions are again reasonably well demarcated. One particularly interesting feature in Fig. 5b (which was alluded to earlier) is that, for both E≤5 (red) and N,E≤5 (black), the normalized mean wealth is positive, while the normalized environmental order is negative. Thus, the dynamics in these regions produces higher wealth than in the non-adaptive (S = 1) case, while producing a *less* ordered environment. Finally, as is apparent from Fig. 4, switching rate is highest in region C (red), quite low in region B (black), but also relatively high in region A (blue). (For examples of the histograms of p(e) and the values of environmental order and mean wealth as a function of time in runs in these three different regions, see Supporting Information S1 and S2.)

**Figure 6:**
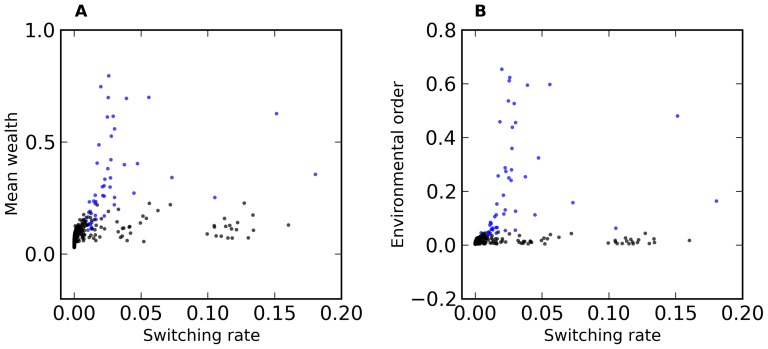
a. Mean wealth vs. switching probability, color coded according to E and N. Red is E≤5 and N>5, Blue is N≤5 and E>5, violet is N and E both ≤5, and black is N and E both >5. Data is the same as that used in Figs. 1–4. **6b. Environmental order vs. switching probability color coded according to E and N.** Same coloring scheme as fig. 6a.

It is clear from these figures that the high switch rate in region C does not result in high mean wealth, nor in high environmental order. However, the situation is markedly different in region A. Refer to Figs. 6 in which we plot switch rate vs. mean wealth (Fig. 6a) and vs. environmental order (Fig. 6b) for runs in regions A and B. (Note the switching probabilities in these regions are less than about 0.2. Switching probability in region C is much higher and is not shown on this graph.) Here we see that mean wealth and environmental order are nearly independent of switching rate in region B, but strongly and positively dependent on switching probability in region A. These observations will be discussed below.

## Discussion

### A. The three regions

The three regions discussed above are associated with very different dynamics. Turn first to region A (N≤5). Here mean wealth and environmental order are both high, suggesting that the co-adaptive system has been able to find reasonably good solutions, marrying high wealth with high order, as we would expect from eq. (3). One interesting, somewhat counter-intuitive feature of this region is that switching rate is still fairly high. Thus, while the system does well at generating mean wealth and environmental order, specific solutions are not generally stable and the system continues to explore other regions of its strategy space.

In region B, (N,E>5) mean wealth is smaller, but still positive, and normalized mean wealth is higher than in region A. Environmental order is small, but non-zero, but is typically *smaller* than in the S = 1 (non-adaptive) case, as seen in Fig. 3b. This is also counter-intuitive: Compared to the S = 1 case, mean wealth is higher, but environmental order is lower. In some sense, the adaptive system, in region B, becomes less efficient at niche formation (lower environmental order), but is very efficient at exploiting those niches which are produced (higher average wealth). The precise dynamical reasons for this are unclear. However, we do believe that the separatrix between regions A and B (which we have estimated here as N = 5) is related to a satisfiability transition

In both regions A and B, it is possible to show, by explicit enumeration, that there are typically very good solutions (i.e., choices of strategies by the agents) which result in very high wealth as well as very high environmental order. However, those solutions are apparently much easier to find in region A than in region B, making this boundary analogous to an easy-hard boundary in a satisfiability problem. From a dynamical perspective, the low switching rate in region B suggests that the system finds a sub-optimal solution and consequently has trouble exploring other regions of the strategy space, in contrast to the dynamics in region A. The work of Thompson [5] who emphasizes the role of co-evolution in crossing maladaptive fitness valleys is also relevant here. The specific boundary between regions A and B depends on S, and also depends on the particular way in which co-adaptation is implemented. Nonetheless, we expect, generically, that there will be two regions with the characteristics of regions A and B.

Because the dynamics and the nature of the typical resulting states in regions A and B are very different, a more detailed study of the boundary between these regions, in particular, how that boundary depends on N, E and S will be illuminating. (In a related model, to be discussed elsewhere, we have found that the boundary between regions A and C is approximately of the form NE = g(S), where g is an increasing function of S.) E can be thought of as a proxy for the degree of potential variation in the environment, while S can be thought of as a measure of the degree of potential adaptivity of the agents. Given a specific method of co-adaptation, the dependence of the boundary on N, E and S may suggest some general principles of the efficacy of co-adaptation in various social and biological (or ecological) systems.

We turn now to region C. The hallmark of this region is high switching rate, low mean wealth and very low environmental order. The dynamics here can be described as thrashing. It is not difficult to show that the probability that at least one of an agent's strategies shares the same reward column with its highest ranking strategy is, roughly,
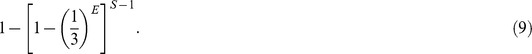
(9)When E is small, this probability can be substantial. Consequently, when it is an agent's turn to act, he will choose randomly among those several highest ranking strategies. This will generate additional randomness in the output states and will consequently lower the environmental order and make it harder for agents to generate wealth. From (9), it is easy to see that E*, the value of E for which, we should find a transition from region C to region A or B should go like
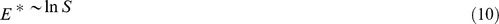
(10)a relationship that simulations validate (Supporting Information S3).

Note that while the switching rate is high both in regions A and C, the nature of that switching in different. In region A the high switching rate is apparently an interesting collective effect as the system explores other regions of strategy space in search of a better solution. In region C, on the other hand, the high switching rate is an individual effect resulting from strategies within an agent that are too similar, and consequently giving rise to thrashing behavior of the agents. This will be discussed in more detail elsewhere.

### B. Induced inter-agent interactions

**Figure 7:**
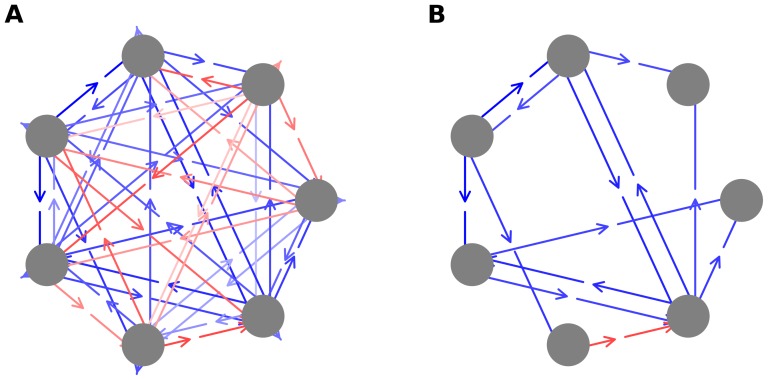
a. An example of an induced agent interaction network for N = 7, E = 5 and S = 16. This interaction network was generated by averaging interactions over a 20,000 time step window. Blue lines indicate positive wealth provided by one agent to another, in the direction of the arrow, and red lines indicate negative wealth. Color intensity indicates the magnitude of the (positive or negative) wealth. **7b. Same network as shown in** **Fig. 7a** **, including a threshold.** In this figure, only lines that exceed a threshold value of ±0.5 points per time step are drawn.

Suppose agent Y acts at time t. His payoff will be determined by the current state of the environment (in the context of the strategy he is using). That environmental state was produced by the agent (say, X) who acted at time t-1. Thus, there is a (directed) induced agent-agent interaction between X and Y, (stigmergically) mediated by the environment. Because agents act in random order, all ordered pairs of agents will have this induced interaction. It is possible to represent this by a fully connected graph in which there are directed pairs of links between each pair of agents, an example of which is shown in Fig. 7a for N = 7, E = 5 and S = 16. This graph represents average wealth that an agent gains from all other agents, averaged over 20,000 time steps. Red indicates net negative wealth, blue, net positive wealth and the density of the color indicates the size of the wealth. While this graph is fully connected, one can place threshold values on the wealth, removing links that fall below a given threshold. The graph will then not be fully connected, an example of which is shown in Fig. 7b. Many interesting questions can be asked about these networks of induced interactions. In general, one can study the distribution of typologies of interactions. Do we induce mutualism or parasitism or other kinds of inter-agent interactions, and what are the distributions of these interactions? (In Fig. 7b, for instance, we see examples of mutualism, commensalism, and amensalism.) Are there agents with special characteristics? For example, one can ask whether there are many agents who are “freeloaders”, who gain wealth from many other agents, but fail to provide positive wealth by their actions. More generally, one can also ask about the distribution of wealth received by an agent from other agents. Do agents primarily receive wealth from only a subset of other agents, or is there typically a wide distribution of wealth accrual by a given agent from other agents. How do these metrics differ in the three different regions of the (N,E) space. These and other related questions will be discussed further elsewhere.

### C. Extensions of the Model

There are a number of variations of this model that beg to be explored. Two of the most important are these: First, the model can be generalized by introducing spatial degrees of freedom. The most straightforward way to extend the model is to add a column to the strategies containing a “move” command. It is unclear whether such models will develop spatially separated niches or sets of distinct environments, and whether the introduction of spatial degrees of freedom will affect the three regions discussed in the zero dimensional model. Second, the model can easily be converted to a model of co-evolution in which the agents' strategies are allowed to change [6]. Using extensions of this model, one can study the dynamics associated with the replacement or mutation of poorly performing strategies, or one can study the dynamics associated with the spread of very good performing strategies (or agents), through sexual or asexual reproduction. (Preliminary studies of a co-evolutionary version of the stigmergy game to be reported elsewhere, indicates a much improved system-wide performance over the co-adaptive system. The improvement may be associated with dynamics described by Thompson [5].) Studying variants of the model will also sharpen the question of what aspects of the model's emergent properties are universal. An important corollary to this study is the confrontation of the results of these models with data. For example, our results suggest that small groups of agents (small N, region A) often have an easier time coordinating and gaining wealth that do larger groups (region B), and, moreover, that they do that in a more actively adaptive way, altering their strategies more often than do larger collectives. Are there examples of social or biological systems that display this property, and how common is it?

### D. Relation to other work

There are at least three distinct bodies of work that are related to our model.

First, in the stigmergy game, we do not specify, *a priori*, the nature of the interactions among agents. Rather, those interactions emerge from the meta-dynamics of co-adaptation. In spirit, this approach is similar to recent work by Worden and Levin [7] and by Akcay and Roughgarden [8]. Those groups, in a game theoretic context, discuss evolutionary models in which, not only do the populations of agents evolve, but also the nature of the interactions among agents and their ensuing rewards also changes over time. In the game theoretic context, this amounts to allowing the payoff matrix governing the interactions among the agents to evolve or adapt, along with the strategies of the agents themselves. In our model, agent actions and their rewards both change as the agents adapt and choose different strategies to play. Another body of work closely related to our model concerns the subject of niche construction [3] [4]. This work emphasizes the importance of endogenous modifications to the environment by the very agents who utilize that environment. Our model, relying as it does on the strong feedback between accrued agent wealth and the structure of the endogenously produced environment, is entirely in the spirit of this approach. For example, one of the questions addressed in Ref. [3] concerns the distribution of acquired cultural traits in a population. The distribution of transition probabilities among agents in our model, i.e., the probability that an agent will produce state e_2_ upon seeing state e_1_ is a direct analogue to this question.

Second, there is a large body of work on models incorporating stigmergy. Stigmergic interactions have been studied in the context of biological systems, such as ant colonies and other social and biological systems including collective projects on the internet [9] [10] [11]. Stigmergic interactions have also been exploited as control mechanisms in various distributed, engineered systems [12] [13]. In much of this prior work, stigmeric interactions are inserted into systems with a preexisting structure, in which agents have a pre-specified set of motivations or actions. Unlike this prior work on stigmergy, we seek to place stigmergic dynamics in a context which is otherwise, as random and structureless as possible to understand what emerges from co-adaptive agents communicating only through the medium of the environment.

Finally, from a formal perspective, this model has some relation to the Minority Game. This is apparent in the structure of the strategies that we use to formulate our model. However, this model differs in some important ways from the Minority Game: Notably, there is no strict limit on the utilization of a limited resource in the stigmergy game. Nevertheless, there are some important similarities between the stigmergy game and the minority game, particularly, the minority game with private information [14]. (In the classic Minority Game agents all respond to the same signals, i.e. to publically available information. In the stigmergy game, agents rank their strategies on the basis of *private* information—i.e., how well each agent did in the past, given the environmental states that agent saw, and regardless of the wealth accrued by other agents.)

In particular, the thrashing dynamics in region C is not unlike the Minority Game dynamics in the mal-adaptive phase, and for a similar reason, namely, sets of agents' strategies are too similar [15] [16]. But there is an important difference. In the classic Minority Game, strategy similarity is *across* agents, whereas in this version of the Stigmergy Game, relevant strategy similarity is similarity in the reward structure and is *within* an agent. In either case, the system-wide behavior is maladaptive due to thrashing, but in the Minority Game, mal-adaptive behavior is accompanied by a system-wide replica-symmetry breaking transition which is not the case in this version of the Stigmergy Game. Another interesting similarity is that in the Minority Game, for fixed N, average accrued wealth, as a function of the amount of information the agents use to make their decisions generally increases as the maladaptive region is approached (i.e., as the number of signals to which the agents must respond decreases). This is very similar to the ridge in Fig. 1a that appears as E decreases toward region C.

### E. Conclusion

The meta-dynamics of co-adaptation or co-evolution is a common feature of complex adaptive systems. In this paper we have taken a first step in an ongoing project of understanding the necessary consequences of co-adaptation or co-evolution. We have studied an *a priori*, relatively featureless model and we have shown how co-adaptation can engender the emergence of a structured environment, positive agent wealth with relatively uniform wealth distribution and induced inter-agent interactions with various characteristics such as symbiosis or parasitism, even when these features are not introduced *ab initio*. The behavior of our model in its three fairly distinct regions also raises a number of other interesting questions, including more detailed questions about the nature of the emergent dynamics in these three regions. In addition, a wide range of other interesting questions, such as the behavior of co-adaptive models in spatially extended systems, can be addressed with straightforward extensions of the model discussed here.

The ways in which structured environments arise, why inter-agent interactions have the characteristics they do, and which of these features necessarily flow from the meta-dynamics of co-adaptation and which are contingent on the details of specific systems are questions that lie at the heart of a better understanding of complex adaptive systems.

## Supporting Information

Supporting Information S1
**Environmental order and mean wealth per agent as a function of time.** In this graph we plot the environmental order,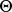, and the mean wealth per agent, W, as a function of time for three runs, one each from the three different regions of the (N, E) plane identified in the text. N = 4, E = 16 (blue), N = 16, E = 16 (black) and N = 16, E = 4 (red). The cross hatched areas of the relevant color indicate the values of 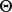, and W to be expected 99% of the time if the environmental states occurred randomly and with equal probability.(TIFF)

Supporting Information S2 **Histogram of the occurrence of environmental states.** In this figure we show the histogram of the occurrence of environmental states, p(e), for the three examples shown in figures S1. Here the histograms are computed from the final N×1000 time steps of each game. The grey bars indicate the range of values for p(e) to be expected 99% of the time, if the environmental states occurred randomly and with equal probability. We also show the location of the three runs in the (N, E) plane.
(TIFF)

Supporting Information S3 **Switching Probability for agents.** Here we show the probability of an agent switching his strategy when he acts, as a function of E for N = 20 and for different values of S. Results are color coded and each dot represents the results of one run. Mean over runs is indicated by the solid line. Note that the s-shaped curve moves to the right like lnS.
(TIFF)
