# Correction: Co-Adaptation and the Emergence of Structure

**DOI:** 10.1371/annotation/b0213eb8-ed75-4885-8063-17524378112d

**Published:** 2013-09-20

**Authors:** Robert Savit, Maria Riolo, Rick Riolo

There were errors in the author affiliations. The correct affiliations are available below.

Robert Savit

Department of Physics, University of Michigan, Ann Arbor, Michigan, United States of America

Robert Savit, Rick Riolo, Maria Riolo

Center for the Study of Complex Systems, University of Michigan, Ann Arbor, Michigan, United States of America

Maria Riolo

Department of Mathematics, University of Michigan, Ann Arbor, Michigan, United States of America 

