# Supplementary figures and images for: Co-Adaptation and the Emergence of Structure
Source: PLoS One. 2013 Sep 10;8(9):e71828. doi: 10.1371/journal.pone.0071828 (PMC3769280; doi:10.1371/journal.pone.0071828)

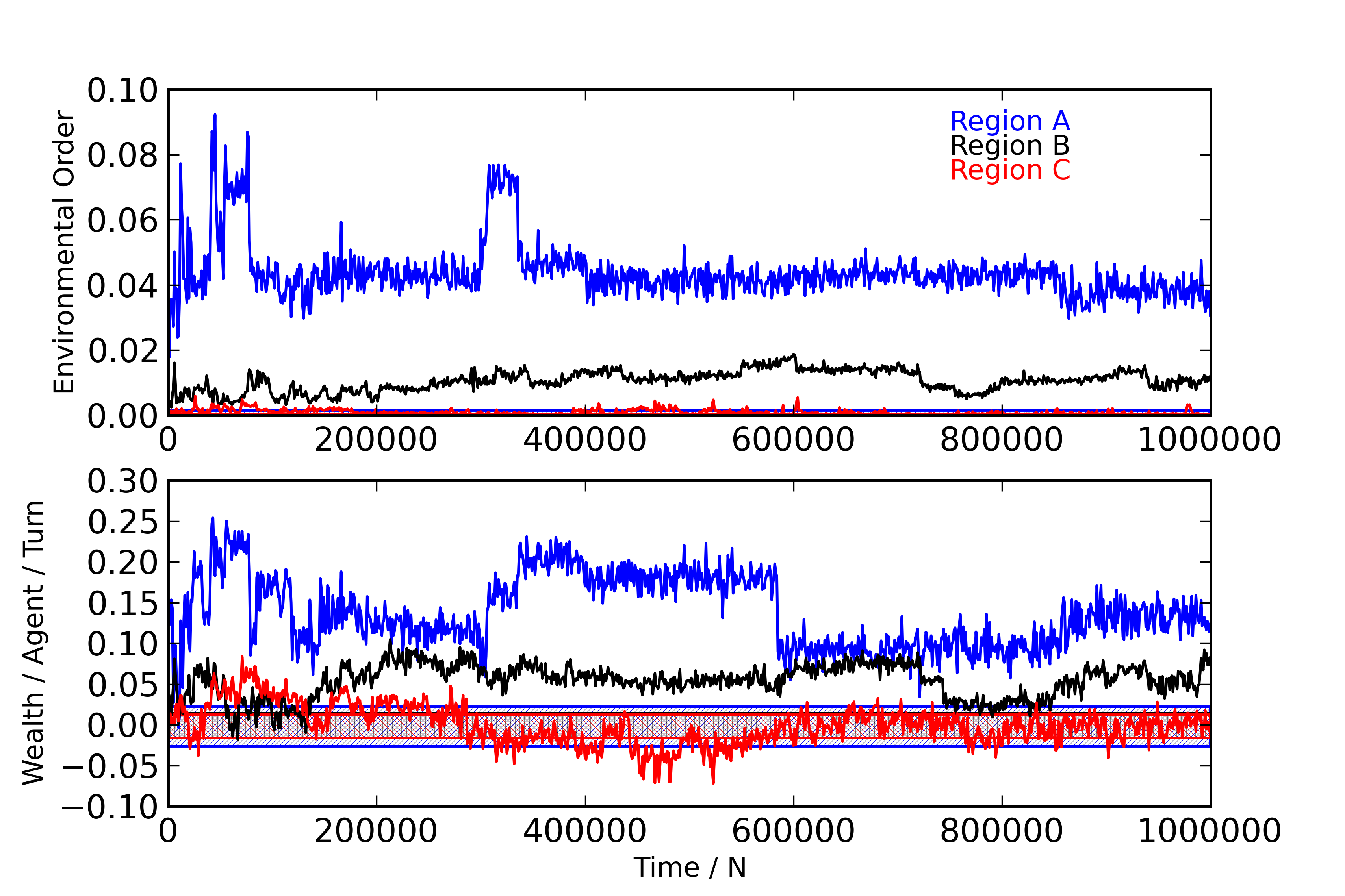

Supplement: Supporting Information S1 — Environmental order and mean wealth per agent as a function of time. In this graph we plot the environmental order,, and the mean wealth per agent, W, as a function of time for three runs, one each from the three different regions of the (N, E) plane identified in the text. N = 4, E = 16 (blue), N = 16, E = 16 (black) and N = 16, E = 4 (red). The cross hatched areas of the relevant color indicate the values of , and W to be expected 99% of the time if the environmental states occurred randomly and with equal probability. (TIFF) [file pone.0071828.s001.tif]

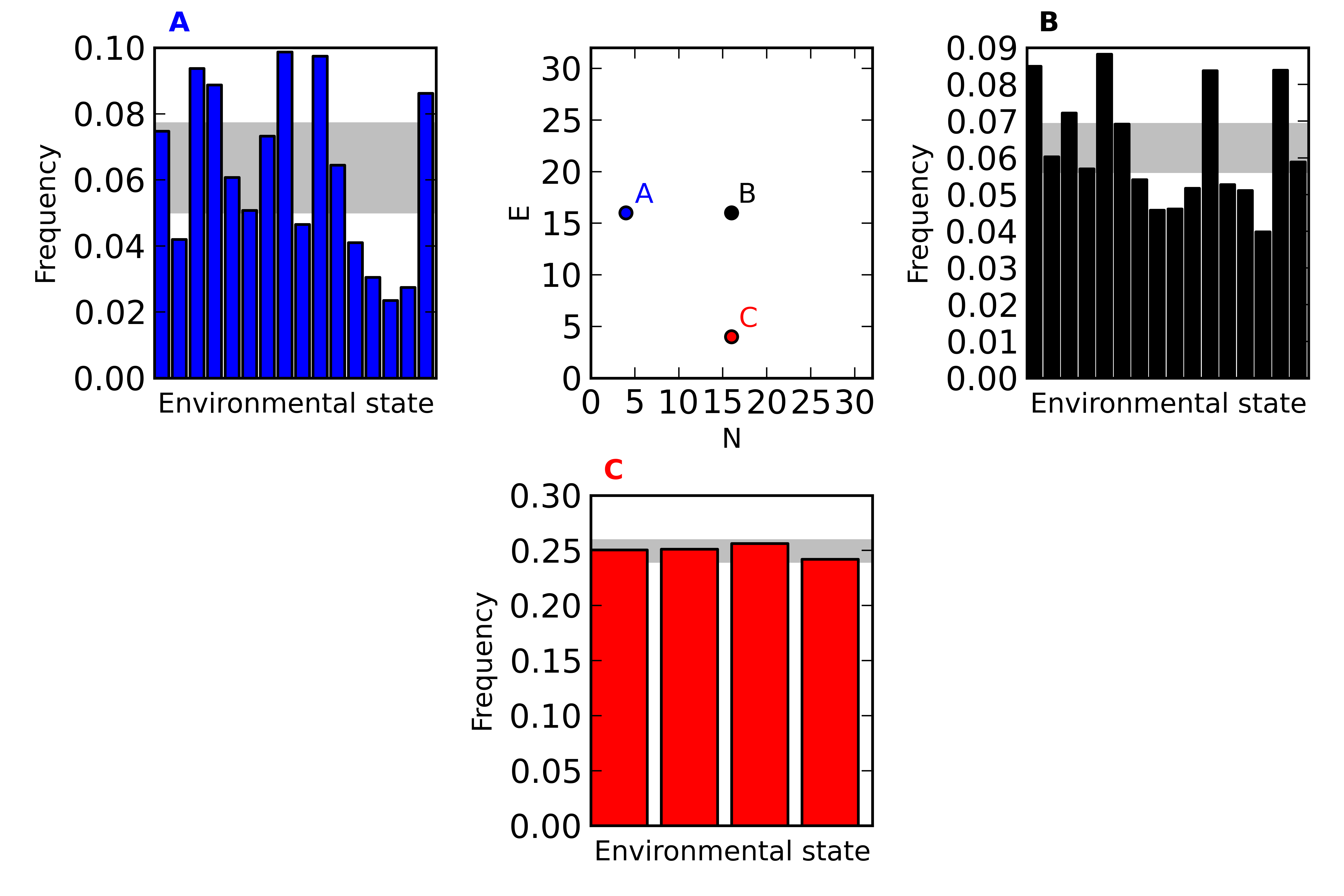

Supplement: Supporting Information S2 — Histogram of the occurrence of environmental states. In this figure we show the histogram of the occurrence of environmental states, p(e), for the three examples shown in figures S1. Here the histograms are computed from the final N×1000 time steps of each game. The grey bars indicate the range of values for p(e) to be expected 99% of the time, if the environmental states occurred randomly and with equal probability. We also show the location of the three runs in the (N, E) plane. (TIFF) [file pone.0071828.s002.tif]

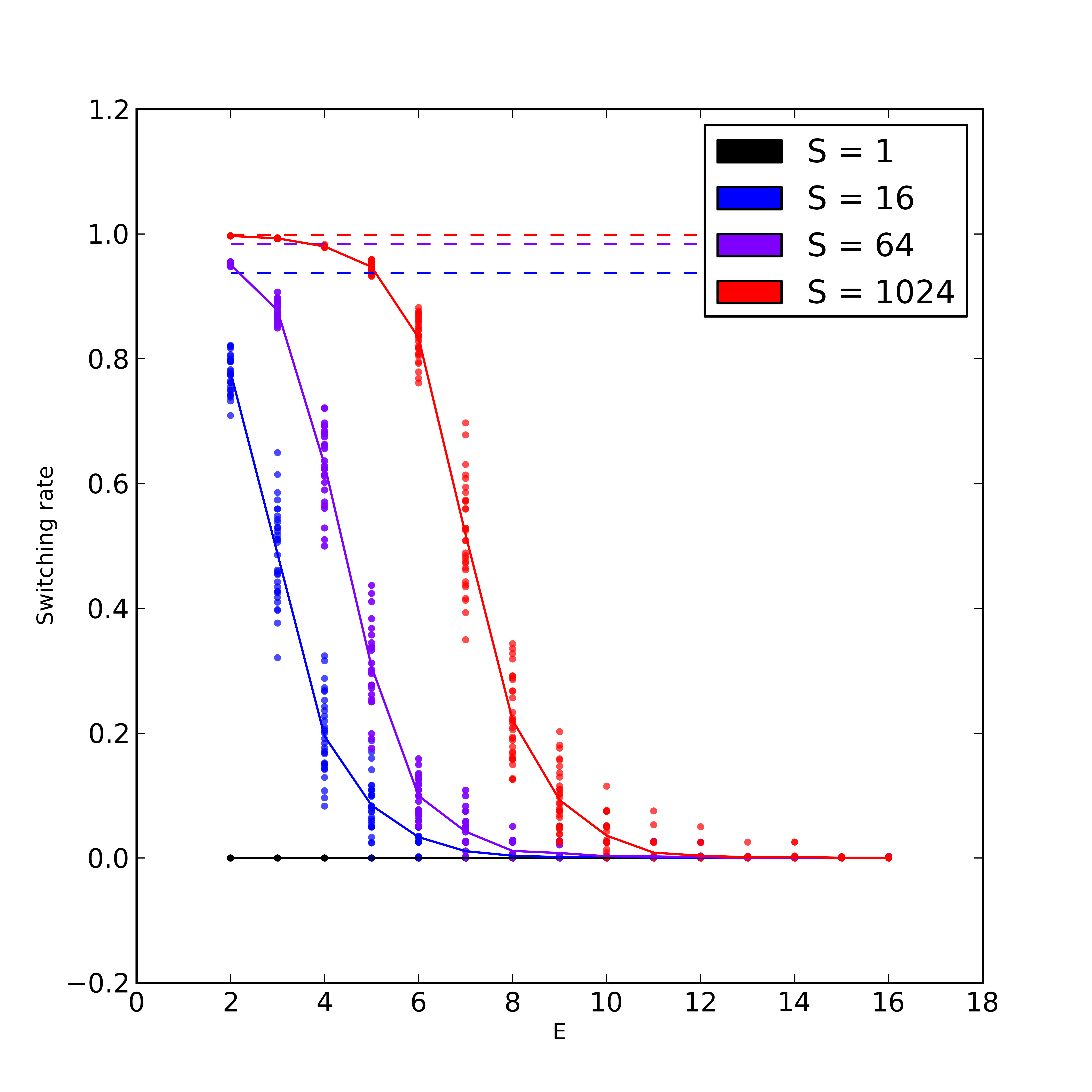

Supplement: Supporting Information S3 — Switching Probability for agents. Here we show the probability of an agent switching his strategy when he acts, as a function of E for N = 20 and for different values of S. Results are color coded and each dot represents the results of one run. Mean over runs is indicated by the solid line. Note that the s-shaped curve moves to the right like lnS. (TIFF) [file pone.0071828.s003.tif]
